# What’s Sex Got to Do With COVID-19? Gender-Based Differences in the Host Immune Response to Coronaviruses

**DOI:** 10.3389/fimmu.2020.02147

**Published:** 2020-08-28

**Authors:** Nirupa Gadi, Samantha C. Wu, Allison P. Spihlman, Vaishali R. Moulton

**Affiliations:** ^1^Division of Rheumatology and Clinical Immunology, Department of Medicine, Beth Israel Deaconess Medical Center, Harvard Medical School, Boston, MA, United States; ^2^School of Medicine, Boston University, Boston, MA, United States

**Keywords:** coronavirus, SARS-CoV, COVID-19, sex, gender, immune response, infection immunity

## Abstract

The novel severe acute respiratory syndrome coronavirus 2, the cause of the coronavirus disease 2019 (COVID-19) pandemic, has ravaged the world, with over 22 million total cases and over 770,000 deaths worldwide as of August 18, 2020. While the elderly are most severely affected, implicating an age bias, a striking factor in the demographics of this deadly disease is the gender bias, with higher numbers of cases, greater disease severity, and higher death rates among men than women across the lifespan. While pre-existing comorbidities and social, behavioral, and lifestyle factors contribute to this bias, biological factors underlying the host immune response may be crucial contributors. Women mount stronger immune responses to infections and vaccinations and outlive men. Sex-based biological factors underlying the immune response are therefore important determinants of susceptibility to infections, disease outcomes, and mortality. Despite this, gender is a profoundly understudied and often overlooked variable in research related to the immune response and infectious diseases, and it is largely ignored in drug and vaccine clinical trials. Understanding these factors will not only help better understand the pathogenesis of COVID-19, but it will also guide the design of effective therapies and vaccine strategies for gender-based personalized medicine. This review focuses on sex-based differences in genes, sex hormones, and the microbiome underlying the host immune response and their relevance to infections with a focus on coronaviruses.

## Introduction

Infecting both wild animals and royalty, the novel coronavirus has been able to proliferate and cause the worst pandemic of the 21st century. As the world races to analyze the behavior of this pathogen and develop a therapy for its disease, epidemiological studies have shown a male sex-based bias in disease severity (1, 2) and increased rates of mortality in men over women (Table 1). A study of over 70,000 coronavirus disease 2019 (COVID-19) patients in Italy revealed a wide variability in the case fatality rate (CFR). Increasing with age, average rates ranged from 0.16 to 20.88% for women and 0.27–34.68% in men. Overall, men were calculated to have a risk ratio up to 1.74 when compared to women. Of course, sex is intimately tied with demographics and characteristics such as gender, profession, and hygiene, and there is a plethora of confounding variables between sex and COVID-19 severity. Men are known to smoke more than women and have higher rates of non-communicable diseases, such as type II diabetes and hypertension (1). Meanwhile, women are more likely to work in the healthcare field and therefore have higher rates of nosocomial infection. However, these factors do not deny the fact that physiology may differ dramatically between the sexes, especially in the context of infection.

**TABLE 1:** Gender-based differences in COVID-19 case and mortality rates.

Country	Cases (%)		Mortality (%)		Total	
			
	Men	Women	Men	Women	Cases	Deaths
United States	48	52	54	46	5,416,639	170,194
Brazil	55	45	58	42	3,340,197	107,852
India	76	24	73	27	2,647,663	56,757
South Africa	43	57	53	47	587,345	11,839
Peru	56	44	71	29	535,946	26,281
Mexico	53	47	65	35	522,162	56,757
Colombia	53	47	64	36	468,332	15,097
Chile	53	47	60	40	387,502	10,513
Spain	43	57	57	43	359,082	28,646
Iran	57	43	59	41	345,450	19,804
United Kingdom	43	57	57	43	275,200	42,072

*Data are collated from the following sources. (1) https://globalhealth5050.org/covid19/sex-disaggregated-data-tracker/; (2) http://www.ijmr.org.in; (3) https://coronavirus.jhu.edu/map.html; (4) https://www.duna.cl; (5) https://www.cdc.gov/mmwr/volumes/69/wr/mm6924e2.htm.*

Across species, females tend to develop a stronger innate and adaptive immune response to contagions. In male and female mice with SARS, male mice had a ∼90% mortality rate, while female mice had a mortality rate of 20%. Doubling the infection load killed every male mouse, while 40% of female mice survived. This overall sex bias was statistically significant and consistent over other strains of mice (3). From an evolutionary standpoint, this increases the reproductive fitness of a species, as mothers are more likely to survive and care for their offspring. Interestingly, parental responsibility is associated with greater immune capability beyond female sex. In seahorses and other fish species, for example, the father is responsible for carrying, delivering, and supporting seahorse fry, and there is an observed upregulation in immunity in these species (4).

Paradoxically, the increased immune function observed among women is accompanied by an increased risk of inflammatory and autoimmune diseases (AD). Women can be 8–9 times more likely to develop an AD compared to men (4). In SARS, these strong immune tactics may inadvertently cause disease through destruction of host tissue. Fortunately, upregulation of proinflammatory immune processes does not seem to occur as much in women with COVID-19. In this case, increased immune function pertains to enhanced anti-inflammatory regulation and antiviral defense (1, 2).

**FIGURE 1:**
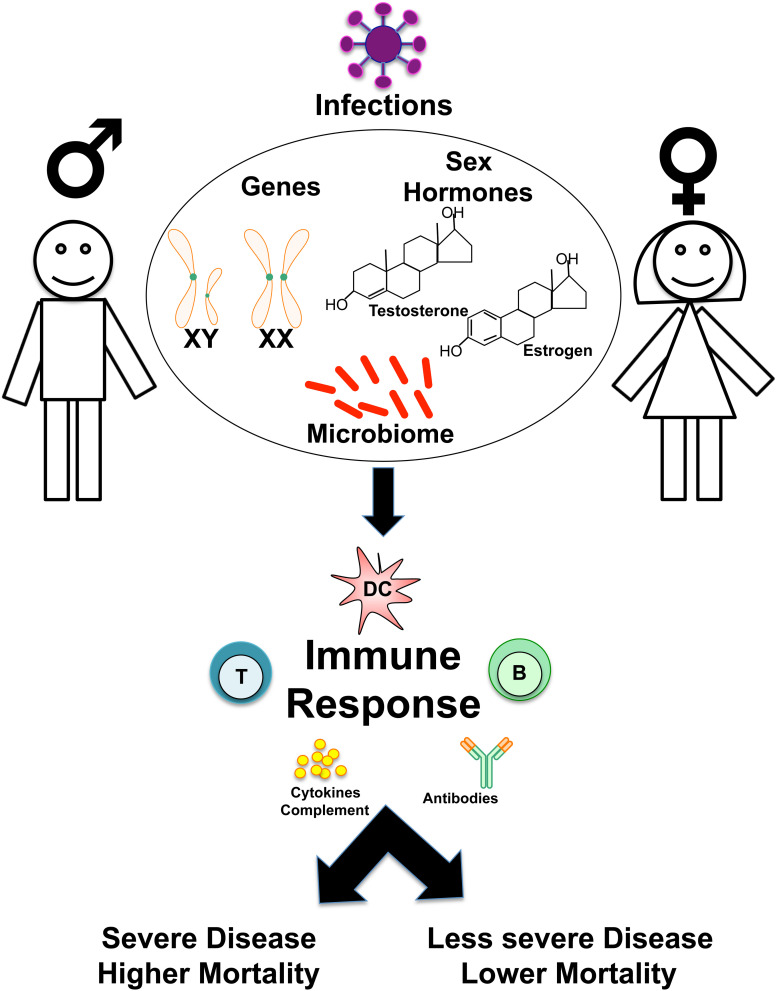
Overview of sex-based differences in the immune response to Infections. Schematic shows how genes, sex hormones, and microbiome may influence sex-based differences in the host immune responses to infections, determining susceptibility, disease course, and clinical outcomes.

**FIGURE 2:**
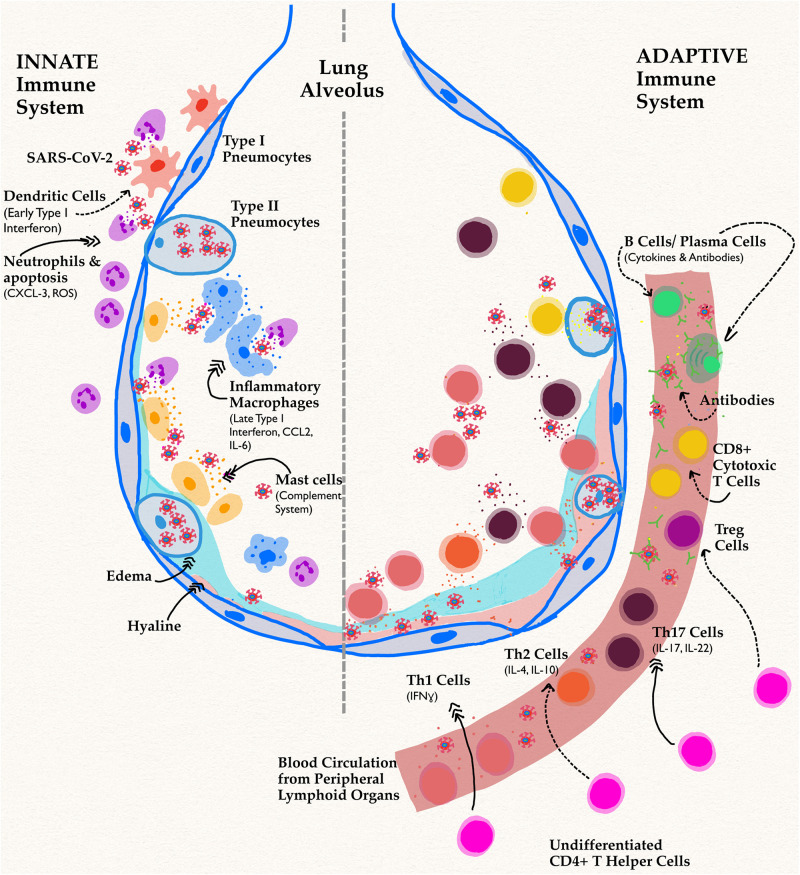
Innate and adaptive immune responses in SARS-CoV-2 Infection. Schematic shows a lung alveolus and a blood vessel. The left side of the figure describes effects of the innate immune system specifically in men, while the right side depicts their delayed adaptive immune functions. Dotted arrows represent a decrease in function or activity, while triple headed solid arrows signify an increase compared to infection in women. The most implicated cell types and their associated cytokines have been shown. In men, the novel coronavirus infection has reduced activation of APC and early proinflammatory processes. A decrease in this pathway causes delayed, excessive proinflammatory cytokine responses leading to the infamous cytokine storm implicated in both components of immunity.

**FIGURE 3:**
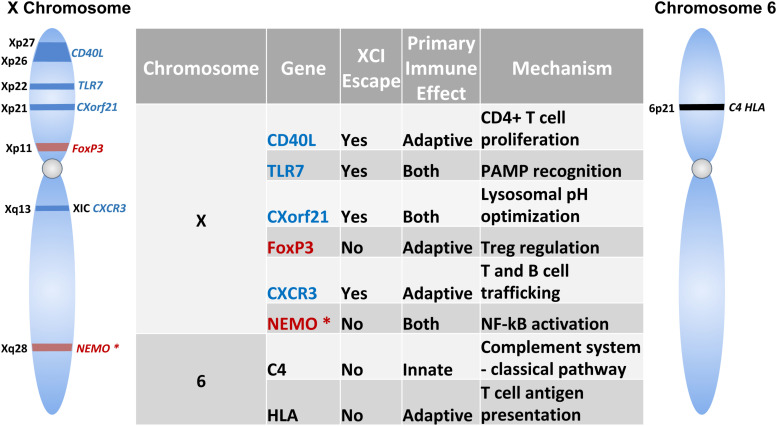
Immune-related Genes implicated in the sex-based differences in the immune response. Schematic shows genes encoded on the X-chromosome and chromosome 6. Table lists the role of these genes in innate and/or adaptive immunity and mechanism of action. Genes indicated in blue text escape X chromosome inactivation, while those that do not undergo escape are indicated in red. NEMO is marked with an asterisk (*) to specify skewed X chromosome inactivation.

**FIGURE 4:**
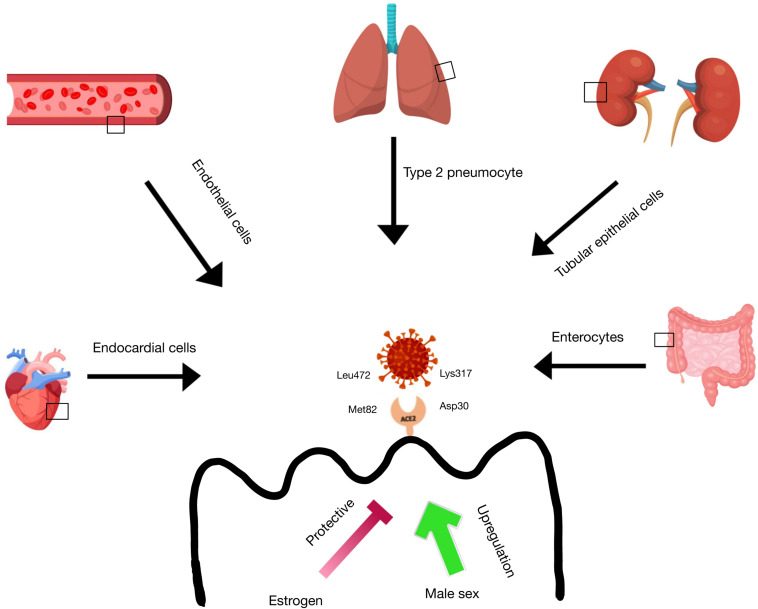
ACE2 receptor expression in tissues. ACE-2 receptor expression is found in the lung type 2 pneumocytes, heart endocardial cells, blood vessel endothelial cells, kidney tubular epithelial cells, and intestinal enterocytes. There is evidence that estrogen has a protective function on ACE-2 receptor expression in these tissues, while male sex is correlated with an upregulation of ACE-2 receptors in these tissues. SARS-CoV-2 is able to bind to the ACE-2 receptor via ionic interactions between Lys317 on the virus and Asp30 on the receptor and via Van der Waals interactions between Leu472 on the virus and Met82 on the receptor.

This review will focus on the intrinsic differences in cell types and humoral components of innate and adaptive immunity (Figures 1, 2). Then, the influence of genetics (Figure 3), sex hormones (Table 2), and microbiome variances across sex will be evaluated. Additionally, the aspects of the ACE2 receptor (Figure 4) for severe acute respiratory syndrome coronavirus 2 (SARS-CoV-2) will be considered, as this unique receptor has multidimensional differences between men and women. Finally, sex differences in the immune response to vaccines will be discussed. Sex is an underappreciated biological variable, and sufficient study and analysis of physiological dimorphisms are necessary to obtain a proper understanding for SARS-CoV-2 interactions in the body.

**TABLE 2:** Sex Hormones and their effects on immunity and relevance to COVID-19.

Hormone	Immune Cell/Cytokine	Effect	Relevance to COVID-19
Estrogen	Type 1 IFN	Promotes synthesis	Proinflammatory, beneficial early on but harmful when delayed
	IL-12	Promotes synthesis	Th1 cytokine, proinflammatory
	IL-6	Promotes synthesis	Pro-inflammatory (cytokine storm)
	IL-1β	Promotes synthesis	Pro-inflammatory (cytokine storm)
	Neutrophils	Delays apoptosis	High recruitment and subsequent apoptosis are found in severe patients
	B cells	Promotes activation, maturation, differentiation, Ig antibody production	Beneficial IgG response but cytokine response is higher in women
	CD4 +	Promotes activation, Th1 differentiation	Different T cell types are needed for successful infection control
	Th17	Suppresses response	Th17 is proinflammatory, decreased levels means less host damage
	CD8 +	Increases activity	High levels early on may confer benefit
	Tregs	Increases FoxP3 expression and Treg production	Tregs suppress Th1 and Th17 responses and are anti-inflammatory
	IL-10	Promotes synthesis	Anti-inflammatory, suppresses cytokine synthesis and MHC expression
Progesterone	IL-1β	Suppresses activation	Th1 cytokine, pro-inflammatory
	TNF	Suppresses activation	Pro-inflammatory, neutrophil and endothelial cell immune activation
	T cells	Decreases proliferation	May control T cell responses and cytokines
	IL-4	Increases production	Th2 cytokine, promotes Ig response controls T cell proliferation
	Tregs	Increases production	Tregs suppress Th1 and Th17 responses and are anti inflammatory
	Th17	Decreases production	Protects the host from adverse immune response
	CD8 +	Reduces IFN-γ production and cytotoxicity	Allows higher numbers of these cells without excess proinflammatory cytokines
Testosterone	TNF	Decreases production	Pro-inflammatory, neutrophil and endothelial cell immune activation
	IFN-γ	Decreases production	Pro-inflammatory, activates macrophages and increases antibody response
	IL-10	Increases production	Anti-inflammatory, suppresses cytokine synthesis and MHC expression

*Table summarizes the role of sex hormones on immune cells and cytokines and the potential relevance to the SARS-CoV-2 infection.*

## Clinical Course

Severe acute respiratory syndrome coronavirus 2 is a novel coronavirus structurally and pathologically related to the original SARS-CoV of 2002. Like its older relative, the new strain of virus accesses host cells using peplomers that are primed by TMPRSS2 to bind the ACE2 receptor (5). The novel coronavirus is an enveloped positive-sense single-stranded RNA virus capable of infecting multiple organ systems in its host, and the density of ACE2 receptors in each tissue correlates with the severity of organ-specific pathology (6). In the lungs, over 80% of cells that express ACE2 are type II pneumocytes, making the lower respiratory tract the most vulnerable target (1). Severe acute respiratory syndrome coronavirus 2 infects these cells and begins processes of viral replication that induce proinflammatory cytokines that recruit components of the innate immune system. The clinical presentations of COVID-19 patients have been heterogeneous, ranging from asymptomatic to respiratory distress to multisystem organ failure and death. One theory to explain this variable response is that the positive inflammatory feedback becomes uncontrolled, resulting in a cytokine storm that can damage host tissue (1, 5). The pathology of COVID-19 disease is characterized by diffuse alveolar damage with fibrin-rich hyaline membranes. Irregular wound healing of the alveoli from the excessive presence of cytokines often leads to thick scarring. This may result in acute respiratory distress syndrome (ARDS) during which patients often require mechanical ventilators, as this lung damage results in restrictive lung disease from fibrosis and pneumonia (1).

ACE2 receptors are also found in extrapulmonary tissues, such as the epithelial cells of the gastrointestinal tract, liver, kidney, pancreas, and olfactory epithelium. They can also be found in cardiomyocytes, pericytes, and fibroblasts of the heart, which may provide a cellular basis for acute myocardial injury. Damage to pericytes and vascular beds may trigger cascades of abnormal clotting, thrombosis, and resultant ischemia that have been noted. Evidence of ACE2 expression in oligodendrocytes of normal brain tissue has also been indicated by RNA-sequencing analyses, which may suggest an explanation for the plethora of neurologic symptoms such as anosmia and ageusia (2, 5). A recent publication reported the clinical characteristics of over a thousand hospitalized adult patients across China. They noted that the most common presenting symptoms were pyrexia (88.7%) and cough (67.8%) (2). Endorsement of congestion, shortness of breath, fatigue, myalgia, headache, and confusion have also been commonly reported in other studies. Emesis, diarrhea, and other gastrointestinal symptoms are also observed in some patients. Brigham & Women’s COVID-19 resource hub reported anosmia and/or ageusia in up to 70% of patients^1^. The complete blood count (CBC) on admission was most notable for lymphocytopenia in 80% and thrombocytopenia and leukopenia in about 1/3 of patients (2). There is a mild hepatocellular injury pattern with AST/ALT ratio ∼200. Elevated d-dimer, CRP, LDH, CK, ferritin, and other markers of inflammation are also commonly reported. Radiographic findings most commonly included atypical pneumonia with bilateral, peripheral, and posterior features. Chest CT findings include ground-glass opacities and bilateral consolidation in more than half of admitted patients (2). Patients with features of these abnormal laboratory findings were most likely in cases of severe disease. Although a plethora of information has been published recently on symptoms, signs, and pathology in COVID-19 disease, little information has been stratified by sex differences. Optimistically, cognizance that these dimorphisms exist will give rise to novel research and therapies that take advantage of these differences.

## Immune Response

### Innate Immunity

#### Antigen-Presenting Cells

Dendritic cells (DC) are the primary antigen-presenting cells (APC) of the human body and are divided into myeloid or lymphoid types based on their visual characteristics. Lymphoid DC are better known as plasmacytoid dendritic cells (pDC) and are the most potent producers of the type I interferon (IFN αβ). Plasmacytoid dendritic cells are activated by several pattern recognition receptors (PRR), of which the most relevant to the topic is Toll-Like-Receptor 7 (TLR7) (7). This PRR is primarily expressed in pDC and recognizes ssRNA sequences, making it vital for detection of coronaviruses in the host (7–9).

The role of IFNα as it pertains to viral infections and SARS in particular has been hotly contested. Many studies show that IFNα has a protective role in Coronaviridae and other viral infections, and its study as a therapy is promising; on the other hand, there is equal literature to show that it is elevated and responsible for adverse host outcomes, such as fibrotic findings in SARS patients (10–12). The current theory behind these conflicting findings is that the timing and level of IFN release is critical to its effect on the host. In a mouse model, Type I IFN (IFNαβ) administration one day after infection seemed to protect mice from adverse outcomes, while delayed IFN exposure enhanced lethal proinflammatory processes (12). Plasmacytoid dendritic cells are responsible for coordinating an early IFN signal, which seems to be associated with better outcomes.

The first major wave of IFNα for antiviral processes is coordinated by TLR7 signaling to pDC (13). This mechanism has been studied to reveal sex-based differences in signaling. For example, pDC from women are found to produce more IFNα when stimulated by TLR7 than compared to men (7). To compound this effect, TLR7 is a receptor that is encoded on the X chromosome and is able to escape X inactivation, meaning that XX females and XXY men [Klinefelter Syndrome (KS)] have higher expression of TLR7 (9). Coronavirus infection magnifies this sex bias, as recent literature has yielded information that SARS coronavirus is a poor inducer of IFN, as its papain-like protease is capable of inhibiting TLR7 signaling to pDC (8). In females, the increased production of IFNα with the enhanced presence of TLR7 is correlated with greater induction of pDC/TLR7-mediated pathways and immune response, suggesting that coronavirus inhibition of host antiviral pathways is reduced (8, 9).

#### Granulocytes

##### Neutrophils

In one study, male and female mice were both infected with SARS-CoV, and their sex-specific outcomes were studied. Male mice had higher rates of vascular leakage, leading to alveolar edema, and a study of bronchoalveolar lavage fluid 3 days after infection showed they had 4–5x higher rates of neutrophils compared to female mice. Neutrophils are vital for a protective immune response, and completely depleting neutrophil populations by use of an αPMN antibody resulted in a 28% mortality compared to a control (3).

However, maintaining a balance in the level of PMNs is critical, as PMNs can also cause pathological states in the host. In the same study, an overly increased presence of PMNs in a coronavirus rat model was found to be correlated with lung tissue inflammation, epithelial cell permeability, and hemorrhagic lesions (14). Additionally, compared with females, male rats’ PMNs, showed significantly higher recruitment of CXCL-1, which recruits neutrophils for killing microbes as well as activating protease and reactive oxygen species (ROS) (11, 15). It seems that in rats, male PMNs have higher rates of apoptosis compared to females, and apoptosis recruits phagocytic cells to the site of cell death (16, 17). Increased damage in the lung tissue of male mice with the presence of up to 500% more PMNs therefore suggests that the neutrophil immune response is pathological in males compared to females, who exhibited few alveolar edema on histological examination.

##### Eosinophils

The eosinophil response in SARS is largely unexplored, especially as it pertains to sex differences. There is, however, convincing evidence that female sex hormones are strong activators of eosinophils. Estrogen promotes eosinophil development, adhesion, and degranulation. Eosinophil numbers spike when female rats have higher estrogen levels, and surgical excision of rat ovaries results in a sharp decrease in uterine eosinophils (17). A genetic study of patients who experienced severe SARS-CoV symptoms revealed that the gene for the enzyme eosinophil-derived neurotoxin (EDN) is expressed more in healthy individuals than in people severely afflicted by SARS (18). Eosinophil-derived neurotoxin is a key protein found in eosinophil granules and has strong ribonuclease activity especially when activated by proinflammatory stimuli. Differences in EDN expression have not yet been analyzed in males vs. females of any species, but if female sex hormones are supportive of eosinophil development and granulation, women expressing EDN may have a unique advantage in fighting SARS infection (12).

##### Basophils

Basophils are the rarest of the granulocytes, and study of the basophil response to coronaviruses is even more unknown than eosinophil interactions. In one study, the incubation of human basophils with mild strains of coronavirus did not cause the leukocytes to degranulate or release histamine (19). More studies are needed, however, to establish that basophils are not involved in the coronavirus response or that there are no sex-related differences in their interactions.

##### Mast cells

Mast cells reside in the submucosal layers of the respiratory tract; although they are mainly known for their functions in allergy responses, mastocytes are also intimately involved in protection from viral invaders (20). In fact, their roles and activation in the immune response are incredibly interesting in the context of sex bias. It seems that, although mast cells from female mice bear granules with higher enymatic activity, these cells are activated to a lesser degree via the complement system than mast cells from male mice. This may explain the increased preponderance of lung injury in men with coronavirus infection. Even when controlling for differences in number and extent of activation, mast cells from female mice make, store, and secrete more histamine than mastocytes of male origin. With binding to high-affinity IgE receptors (FcεR1), mast cells from female mice release an increased amount of other preformed proinflammatory mediators, such as tryptase, chymase, and TNF-α (17, 21). Gene analysis study has shown that genes such as *Tnf, Hexa*, and several *mcpt* genes that encode these intracellular granule mediators are upregulated in female mice. Mast cells from women store this increase in protein product by increasing the packing density of granules in mast cells (22). This set of studies was repeated in the presence of various levels of female sex hormones, as previous literature has shown that the menstrual cycle can affect properties of mast cells in rodent models. The study found that there was no statistically significant difference in the amount of histamine that mast cells released across levels of sex hormones in male and female mice (21). This shows that while previous studies may point to significant hormonal effects on mast cell properties, there are sex-based differences in mastocyte biology that are not attributable to hormone interaction.

Mastocytes in the context of SARS-CoV infection are intimately involved with the complement system, which has significant proinflammatory responses that can result in pathological states. In fact, coronavirus infection activates the classical, the lectin, and the alternative pathways of the complement system. Of particular interest are C3 and C5 proteins which activate mast cell degranulation in SARS to trigger a cytokine storm (19, 23). This cytokine storm can lead to further downstream effects such as hyperemia and vascular permeability, which can result in acute, fatal lung injury. C5a levels are actually predictive for ARDS development, and blockade of the C5a pathway in MERS infection reduces lung injury in mice (20). In a similar vein, inhibition of the complement cascade via inhibition of C3 in M15-infected mice results in less recruitment of neutrophils and inflammatory monocytes to the lung tissue (23).

The conclusion that reduced activation of the complement cascade results in maintenance of healthy lung tissue correlates to the sex bias found in levels of complement proteins. A recent study of 50,000 racially diverse subjects found that, compared to men, women have significantly lower levels of C4, an activator of C3. They were also able to prove that this results in a 42% decrease in C3 in women compared to men (22). In an investigation of Caucasian populations, levels of C3, C5, C7, C8, and C9 were significantly lower in women compared to men. Women had about 53% lower C5 protein levels than men. Also women had lower amounts of proinflammatory positive regulators of the cascade such as properdin, mannan-binding lectin (MBL), and Ficolin-3 (24). These reduced levels of complement proteins were found to control activity of the whole cascade and its terminal products for the classical, lectin, and alternative pathways that are involved in SARS infection (20, 24). While these findings were obtained from a Caucasian cohort, evaluation of these in other race/ethnicities remain to be seen. Yet, from both studies it is justifiable to say that the female host has mechanisms to reduce proinflammatory effects even with more potent mastocytes. In the context of SARS, these techniques may prove useful if they are an explanation for the minimized incidence of pulmonary injury.

#### Monocytes/Macrophages

In the discussion of pDC, it was iterated that the role and clinical effects of Type I IFN are still incompletely understood and are often conflicting across various studies. Current theories support the idea that initial secretion of Type I IFN is effective at reducing viral load without causing damage to the human host in which XX females and XXY males seem to have better outcomes than XY males in a mouse SARS-CoV model. For example, Type I IFN administration 6 h post-infection (before viral peak) in mice infected with SARS coronavirus completely protected them from clinical disease (10, 12). Elevated and extended exposure of the host to IFN, however, led to excessive proinflammatory pathways and pulmonary pathology. In the same study, mice were exposed to IFN post-peak of viral titers which resulted in lethal pathology. This acute lung injury in mice and humans with SARS is characterized by the presence of IFN-stimulated inflammatory monocyte-macrophages (IMM) and their associated proinflammatory cytokines in bronchoalveolar lavage fluid (10–12). Although women have higher activation of IFN pathways early in coronavirus infections, it is unknown if there is a difference in IFN levels between men and women after peak viral load. It may be that early activation of antiviral pathways more effectively reduces viral load by priming the innate and adaptive immune systems. This early activation can better protect the host from the cytokine storm found mostly in males that is associated with later IFN secretion (10, 12, 25).

Although the sex differences regarding late Type I IFN levels are unknown, SARS-CoV-infected male mice have higher rates of IFN-stimulated IMM in their bronchoalveolar lavage fluid. Just three days post infection, there were 2–3-fold greater numbers of these IMM (11). Cytokine analysis showed that IMM release from males had a higher frequency of the pro-inflammatory cytokines CCL2 and IL-6. IL-6 is an activator of CCL2 binding to CCR2 which promotes lymphoid and myeloid chemotaxis as well as properties of leukocyte adhesion, polarization, secretion, and survival in the immune responses (12, 26). However, because this IFN-stimulation of IMM is uncontrolled in SARS patients with poor outcomes, methods of reducing their potency are advantageous. Ablation of IFNAR, a Type I IFN receptor, 3 days into infection produced improved outcomes from coronavirus infections (10). Additionally, use of monoclonal antibodies has also been considered for therapy in humans. In SARS-CoV infection in mice, MC21 antibodies could bind to CCR2, and this competitive inhibition provided significant protection in male mice prone to poor-outcomes. This study demonstrated that a depletion in IMM signaling is a protective feature in SARS-infected female mice (11).

#### Natural Killer Cells

Natural Killer (NK) cells are cytotoxic lymphocytes of the innate immune system that target cancerous and infected cells. Their exhaustion is correlated with disease progression (27), as they are primary secretors of IFN-γ, TNF-α, colony stimulating factors (CSF), and many other cytokines (15, 27). In the diseases caused by both SARS-CoV and SARS-CoV-2, studies have shown that there is a marked decrease in the total number and activity of NK cells in infected patients. In the same vein, there are significantly lower recorded amounts of CD107a+ NK, IFN-γ+ NK, IL-2+ NK, and TNF-α+ NK in COVID-19 patients, suggesting that functional exhaustion of NKC is associated with coronavirus infection (27).

There is a plethora of puzzling information about the sex bias in NK cells. Some studies have found greater numbers and activity of NK cells from male rodents compared with females (4, 17). Another study of healthy geriatric individuals found that while there was an initial surplus of NK cells in men less than 70 years of age, there was a steep increase and superiority in NK cell function in women over 70 years (17, 28). This post-menopausal finding seems to defy other studies that found a rise in NK cells during the periovulatory phase when estrogen and progesterone are increased (4, 29). It is possible, however, that even if estrogen derivatives increase the number of NK cells, the cytotoxic capabilities of these cells may be reduced.

### Adaptive Immunity

#### CD4 T Cells

##### Th1/Th2 cells

The physiology of CD4 T helper type 1 (Th1) and 2 (Th2) cells in COVID-19 infection is the most striking when considering the implications for sex differences. Th1 cells are differentiated CD4+ T lymphocytes that are microbicidal and proinflammatory. In contrast, Th2 cells produce cytokines that are more anti-inflammatory and redirect focus to humoral immunity mechanisms. The homeostasis between Th1 and Th2 cells is vital for the obliteration of infectious microbes without causing pathological states in the host. The Th1/Th2 ratio is paramount for host success, as extermination of distinct pathogens will require different levels of activation in Th1 and Th2 lymphocytes (30–33). An improper ratio of Th1/Th2 cytokines may result in prolonged disease states such as in lepromatous leprosy or tuberculosis.

The SARS coronavirus of 2002 is notable in that it exclusively produced an activation of Th1 cells and cytokines in the host. In a study of patients in China, the Th1 response produced a significant elevation in the proinflammatory cytokines IFN-γ, IL-1β, IL-6, and IL-12 (30). These protein levels were elevated for at least 2 weeks after onset and were sufficient for full recovery of the host. Across several studies, induction of anti-inflammatory Th2 pathways was not necessary for host survival (30–32). The novel coronavirus SARS-CoV-2, however, seems to work differently when compared to its older relative, as sufficient induction of anti-inflammatory Th2 response is necessary. Patients with COVID-19 show activation of both Th1 and Th2 pathways over the course of infection with significant levels of IFN-γ, IL-1β, and IL-6 as well as IL-4 and IL-10, respectively (31, 32). An elevated Th1/Th2 ratio has been correlated to increased risk of mortality in COVID-19 patients as the proinflammatory cytokine IL-6 has been predictive for severe lung pathology (31). Another COVID-19 study found that aberrant Th1 cells expressing significant levels of IFN-γ, IL-6, and GM-CSF were found only in intensive care unit (ICU) patients. Levels of these cytokines were much lower in non-ICU patients and the control group (32), indicating that pathogenic Th1 cells correlate with the hyper-inflammatory response in SARS-CoV-2 pathogenesis.

To the best of the authors’ knowledge, there is no literature yet on sex differences in Th1 and Th2 responses to COVID-19. However, the implications of the Th1/Th2 balance could be important in pregnant women with SARS-CoV-2 infection. Physiologic changes within the immune system during pregnancy typically involve an attenuation of the Th1 response and a shift toward Th2 anti-inflammatory pathways (31, 32). This is in the interest of protecting the developing fetus from an overactive cell-mediated immune response, while simultaneously developing antibodies for passive transfer of immunity through the placenta and breast milk. In the Th1-dominant SARS-CoV infection, the CFR of pregnant women was estimated to be up to 18%, as the maternal immune system would attenuate Th1 and inadvertently compromise itself. The original SARS-CoV stands in contrast to the novel SARS-CoV-2 COVID-19, however, where the CFR is ∼1% (34); although hospitalization, ICU, and ventilation requirements were higher in pregnant compared to non-pregnant women, the risk of death was the same compared to non-pregnant women (35), and disease course appears to be milder than in SARS-CoV and MERS (31). Although there may be numerous variables at play, it seems that host survival in COVID-19 is tied to substitution of the proinflammatory Th1 for the anti-inflammatory Th2 that is dominant in gravid hosts.

##### Th17 cells

Th17 cells are a differentiated form of CD4+ T lymphocytes that mainly produce IL-17, IL-22, IFN-γ, and GM-CSF. They are also involved in the production of related proinflammatory cytokines such as IL-6, IL-26, and TNFα (36–38). It was found that the severity in MERS and both forms of SARS was positively correlated with Th17 and IL-17 levels in patients. IL-17 is the most well-studied cytokine of Th17 cells, as it is the lymphocyte’s hallmark cytokine. In coronavirus infections, IL-17 encourages the assembly of downstream proinflammatory cytokines that result in activation of neutrophil chemokines and secretory elements that damage lung parenchyma (37). An investigation of polymorphisms for IL-17 genes in SARS patients showed that individuals predisposed to lower levels of IL-17 activation had significantly increased 30-day survival when compared with patients prone to increased IL-17 production (36). Th17 lymphocytes also contribute to ARDS pathogenesis by activation of IL-22, which seems involved in the production of mucin and fibrin-rich secretions in patients with pulmonary edema (36, 38).

While the role of gender in relation to Th17 function in coronavirus infections has yet to be studied, there is some literature on how this cell type affects AD. In AD that predominantly affect males, there has been significant literature on Th17 cells playing a paramount role in disease progression (37–39). For example, although multiple sclerosis (MS) affects 2–3 times more women than men, men tend to experience more rapid and aggressive disease progression (39). Similarly, in systemic lupus erythematosus (SLE) which afflicts women 9–10 times more often than men, men tend to experience more severe complications especially nephritis leading to renal failure. In a mouse model of MS, Th17 cells from male mice were transferred into female mice and found to induce significantly higher levels of IL-17 and IFN-γ secretion that led to worsening AD and pathology (38). This finding suggested that male sex is a crucial and inherent element of disease-induced severity related to Th17 cells. Whether this finding is applicable to host attack in the context of SARS remains to be determined.

##### Treg cells

Regulatory T (Treg) cells are a subset of CD4 T lymphocytes with unique immunosuppressive activities. As discussed previously, the homeostasis between proinflammatory/anti-inflammatory processes is critical to clearance of an infection without damage to the host and subsequent AD (40). The optimal balance of these activities is different for every pathogen, but, in the context of mouse hepatitis virus (MHV) coronavirus, Tregs are necessary for mild disease outcomes, as their depletion results in increased mortality (40, 41). While studies of Treg influences and functions have been performed on several types of respiratory viruses, there is little information on their roles against human coronaviruses. As seen in the disparity between Th1/Th2 responses with both SARS coronaviruses, adaptive immunity mechanisms may vary significantly even between similar virus strains. The role and activity of Treg lymphocytes, therefore, cannot yet be verified in the context of COVID-19, but these cells may offer a benefit by reducing excessive damage to lung parenchyma.

The transcription factor Foxp3 serves not only as a marker for Treg cells, but it is also necessary for their development, maintenance, and suppressive functions (42, 43). Foxp3 is encoded on the X chromosome and can escape X inactivation, giving XX females higher activity of Foxp3 and Treg cells (33, 41). This may give women an immunosuppressive advantage in the context of coronaviruses. Scarcity of Treg cells from loss-of-function alterations in *Foxp3* lead to severe and even lethal inflammation in human and rodent models of coronavirus infection (41). There is more literature on this appreciable sex bias of Treg cells that will be expanded on in the “Genetics” section of this article.

#### CD8 T Cells

CD8+ cytotoxic T cells are differentiated lymphocytes that kill infected, cancerous, or damaged cells through recognition of antigens presented via major histocompatibility complex (MHC) class I and consequent signaling. Across several studies, there are a significant portion of COVID-19 patients who present with lymphopenia and markers of T cell exhaustion. PD1 and Tim3 are molecular markers of this fatigue in CD8+ cells and are elevated with lower levels of circulating cytotoxic T cells. It was found that as patients progressed from prodromal to active symptoms, their levels of PD1 and Tim3 would directly increase. In the same vein, patients in the ICU had much higher expression of these markers compared to non-ICU and control populations (44, 45). Another study used granzyme B and perforin markers of induced apoptosis as indicators of CD8+ cell fatigue. Consistent with previous work, these markers were significantly elevated in critically ill patients compared with those who were only mildly symptomatic with COVID-19 (46). This new data indicates that SARS-CoV-2 promotes extreme stimulation and ensuing exhaustive collapse of CD8 + T cells, which is similar in fashion to many malignancies (44–46).

Causes and effects of lymphopenia have not been thoroughly studied in SARS, and much less can be said for differences in sex. Healthy females in general, however, exhibit higher cytotoxic T-cell activity along with an upregulation of CD8+ genes (47), many of which have estrogen response elements (ERE) in their promoters. Also, as discussed earlier, women have greater activation of TLR7 and pDC. It appears that upregulation of this pathway results in higher levels of CD8 + T-cell activation in women compared to men (7, 13, 47). The implications of greater CD8+ function are unknown in the context of this novel virus, but it may decrease viral load in early coronavirus infection (47).

#### Memory T Cells

After an initial encounter with a pathogen, antigen-specific naive CD4 and CD8 T cells clonally expand to become effector T cells, which mount a cellular and humoral response against the offending pathogen. After pathogen clearance, most effectors die by apoptosis, while some survive and persist to become long-lived memory T cells, and it is these cells that offer the host protection from subsequent encounters with the pathogen. Memory cells also form the basis for vaccinations which elicit a similar immune response albeit at a significantly reduced magnitude without causing disease. Memory T cells are heterogeneous in phenotype, function and localization and include central memory (Tcm), effector memory (Tem), tissue resident memory (Trm), terminally differentiated memory (Temra), and other cells (48). These antigen-specific cells are the basis of vaccine development, as they are skilled in triggering a targeted immune response upon re-exposure to an antigen. As SARS-CoV-2 is a novel infection, development and viability of memory T cells in men and women is truly unknown, especially in the face of viral mutation.

As SARS-CoV-2 is a novel infection, development and maintenance of memory T cells in men and women is truly unknown, especially in the face of viral mutation. Several studies on the original 2002 SARS-CoV have, however, shown that SARS-CoV-specific CD8+ memory cells can be found 6–11 years later in the blood of past patients, while specific CD4+ cells have only been reported for up to 2 years (48–51). In support of a gender difference in memory T-cell survival, it appears that healthy women harbor greater numbers of CD4+ lymphocytes (52). In SARS, however, CD4+ T cells have mainly exhibited a central memory phenotype while CD8+ cells were of the effector memory phenotype, suggesting that CD8+ memory cells may be dominant in host attack upon re-exposure (51). This also suggests that CD4+ abundance in women may not affect long-term and effective resistance to SARS. In anamnestic response of CD8+ cells to the SARS coronavirus, these lymphocytes proved valuable in that they could produce cytokines such as IFN-γ, TNF-α, IL-2, and granzyme B to reduce lung viral loads in mice (49–51). A study did, however, find that while CD8 + cells were functional in protecting vulnerable hosts from reinfection, their pathways were downregulated without SARS CoV-specific CD4+ T cells or differentiated B cells (49).

While memory T-cell immunity may be the key to protect from reinfection from SARS-CoV, SARS-CoV-2 has shown evidence of mutation and therefore carries the possibility of reinfection. Vaccine development has been rapidly under way since the novel coronavirus’s introduction, and recommendations based on genome analysis have been made to target portions of the virus that are least likely to mutate. A promising population coverage analysis found that 23% of known SARS-CoV-2 epitopes map parallel to SARS-CoV, and no alterations have been reported in these epitopes among known SARS-CoV-2 genomes (53). This strongly suggests their potential for inducing a viable T-cell response against SARS-CoV-2.

#### B Cells

##### Antibody functions

Antibody secretion is the primary function of B lymphocytes. In a study of almost 300 patients who had SARS-CoV-2, 94% formed IgM antibody titers, and 100% developed IgG at a median of 13 days post symptom onset (54). As expected, IgG antibody levels were stable while IgM antibodies reached low levels within 5 weeks and became undetectable at 7 weeks (55). Importantly, the IgM response typically precedes IgG, but several investigations have seen synchronous seroconversion of IgG and IgM as well as appearance of IgM or IgG first at about equal rates (54, 55). This may have to do with the upregulation of IL-4, IL-10, and other cytokines promoting antibody class-switching at an ultra-rapid rate (56). These interleukins are part of the Th2 pathway which is naturally enhanced in women, suggesting that there may be a physiological sex difference in antibody switching, although no known studies have been done in this specific area (32) (34). Another study investigated IgG levels between male and female COVID-19 patients, stratifying patients by disease severity into mild, moderate, and severe status and into early, active, and recovery phases. While there was no significant difference in serum IgG levels across gender in mild and recovering patients, IgG levels in women were significantly elevated in the early disease phase and in severe cases (57). This situational increase in antibody titers cannot yet be determined as helpful or harmful in SARS, but it is worth noting for future investigation.

##### Cytokine functions

B-cell production of cytokines make these lymphocytes powerful regulators of adaptive immunity, but in many cases turn maladaptive, such as in SARS. A series of seven case studies was recently published on B-cell immunocompromised COVID-19 patients and sheds light on the excessive lymphocyte immune response. Disease presentation of patients with common variable immune deficiency (CVID) hypogammaglobulinemia were compared to patients with agammaglobulinemia (AGG). Surprisingly, the AGG cases proved to be mild, and the patients had normal lung CT scans with no consolidation. The patients were treated in the hospital for a maximum of 3 days and went home without requiring assisted ventilation. In contrast, the CVID patients had extensive ground glass opacities and alveolar consolidation on lung CT. They were in the hospital for at least two weeks and needed antiretroviral therapy (ART), IL antagonists, and some required mechanical ventilation. These findings suggest that complete depletion of B cells is associated with mild symptoms in SARS. According to the study, the difference in patient outcomes was likely due to the lack of non-Ig B cell cytokine functions, meaning that patients with AGG avoided a host-harming cytokine storm. Based on these results, inhibiting B-cell cytokines may be a useful tactic in both men and women with COVID-19 (47).

This cytokine activation is linked to stimulation of TLR in T-cell independent activation (46). Blood analysis of COVID-19 patients has shown marked decreases in naïve B cells with increases in plasma cells, suggesting that there is a pressure on naïve B cells to mature and proliferate for increased humoral immunity. In the context of coronaviruses, IFN-α is released by pDC upon stimulation from TLR7 binding. This causes an upregulation of TLR7 receptors on the surface of the naïve B lymphocyte. This process authorizes B cells to respond to activation from coronavirus-TLR7 binding by immediate expansion and differentiation without much T cell interaction. These mature and activated B cells are capable of producing IgM as well as proinflammatory cytokines, namely IL-6. IFN-α is also able to induce this effect in memory B cells which are activated to produce IgM and IgG. Together, these data indicate that pDC IFNα controls the proliferation and differentiation of B lymphocytes into Ig-secreting plasma cells. This conclusion is paramount in coronavirus infections, as women express more TLR7, initially secrete more IFN-α, and therefore have greater activation of B-cell antibodies and cytokines (45, 46). Contrary to other immune cells (discussed above), it seems that B-cell-derived cytokines may be more harmful to a female host.

### Genetics

Women mount a stronger immune response against viral infections than men (58). Women possess both maternal and paternal X chromosomes, which necessitates the silencing of one copy of genes in order to ensure an appropriate gene dosage. The silencing of one copy, or X chromosome inactivation (XCI), leads to functional mosaicism in women with regards to X-linked genes (58). The X inactivation center (XIC) is located at locus Xq13 (58). X chromosome inactivation is cell-specific and variable among individuals, which causes some cells to express the maternal chromosomal copy and others to express the paternal copy. In turn, this leads to a diversity of possible immune responses in females, which provides women with a wider variety of tools with which to fight pathogens (59). Skewed inactivation patterns may additionally offer a protective effect by silencing immunodeficiency-causing mutations (60). Furthermore, X-chromosome skewing may preferentially express beneficial alleles, leading to a larger proportion of cells producing functionally advantageous gene products (60). X chromosome inactivation is particularly relevant to discussion of the SARS-CoV-2 immune response, as the X chromosome encodes for several genes involved in both adaptive and innate immunity, including those involved in the TLR pathway (58). Cellular mosaicism suggests that women may be better equipped to respond to immune challenges, particularly viral infections such as SARS-CoV-2.

Several genes fail to undergo XCI and thus “escape” inactivation, leading to biallelic gene expression with a double dosage and resulting differences in gene dosage between sexes. About 15% of X-linked genes escape XCI, while 10% are only partially inactivated (59). Evidence shows that XCI escape commonly occurs in female lymphocytes, which display atypical heterochromatic modification and tend to reactivate the inactivated X chromosome (Xi) (61). Expression of X inactive specific transcript (XIST) RNA has been observed in both B and T cells, leading to biallelic expression of CXCR3, TLR7, and CD40L (58, 61). The XCI escape of genes involved in the immune system may further contribute to an immunologic advantage in women.

#### CXCR3

Chemokine receptor CXCR3, located at locus Xq13, is one such gene that escapes inactivation (58). *In vitro* mouse studies of CXCR3 expression confirm that females express both copies of CXCR3. Biallelic CXCR3+ T cells yield more CXCR3 protein and subsequently secrete more IFN-γ, IL-2, and CD69 than monoallelic CXCR3+ T cells (62). CXCR3 functions to mobilize NK cells and T and B lymphocytes to areas of inflammation and may aid in effector Th1 cell differentiation (63, 64). The role of CXCR3 in leukocyte recruitment and the Th1 response implies that the increased expression of CXCR3 may cause a stronger antiviral response in females (62). Amplified CXCR3 signaling due to XCI escape may thus contribute to increased immune activation and better ability to combat SARS-CoV-2 infection in women.

#### Toll-Like Receptor-7

Toll-like receptor-7 (TLR7)-mediated secretion of IFN-α has been demonstrated to play a key role in the response to coronavirus infections (13). The gene for TLR7 is located at Xp22.3 and participates in XCI escape, leading to overexpression in women (65). About 30% of immune cells from women and 19.3–39% of immune cells from XXY KS men display biallelic expression of TLR7 (66). In the presence of TLR7, CD27+ B cells were found to proliferate more quickly in female cells than non-KS cells, suggesting amplified TLR7 signaling in biallelic cells (66). When stimulated by TLR7, biallelic B cells were 2.4 times more likely to undergo IgG class switching compared to monoallelic B cells (66). Increased class switching suggests that women and KS males may have enhanced humoral immune response due to TLR7 overexpression. Estrogen levels likely contribute to the sex-based difference in TLR7 signaling, as immune cells from both men and women have been shown to increase TLR7 expression post-exposure to estradiol treatment (60, 67). Evidence suggests, however, that biallelic expression of TLR7 may also increase TLR7 signaling capacity. Transplanted pDCs from women produced a heightened TLR7-mediated IFN-α response to influenza virus and HIV pathogen-associated molecular pattern molecules (PAMPS), regardless of the sex of the mouse host (67). This finding implies a contributor to the IFN-α sex bias which is intrinsic to the immune cell. This factor is likely independent from hormone signaling, as similar increases in IFN-α were observed when comparing pDCs from men and women that were transplanted into female mice (67). As a result, the overexpression of TLR7 in biallelic pDCs may play a part in increasing secretion of antiviral IFN-α.

Further studies of 47, XXY karyotype KS—men with an extra X chromosome—and Turner’s syndrome (TS)—women lacking an X chromosome with a 45, XO karyotype—implicate XCI escape in the sex-based difference in the immune response. KS men, but not TS women, typically undergo XCI. As a result, inclusion of genotypically diverse individuals may lend additional insights into the impact of XCI escape on TLR7 gene dosing. Lymphocytes purified from women and KS men expressed more copies of TLR7 mRNA when compared to men and TS women after exposure to TLR7 agonist CLO97 (65). As a result, XCI escape may be correlated with increased TLR7 signaling and more vigorous immune response to viral infections.

#### CXorf21

CXorf21 is located at gene locus Xp21.2 and escapes X-inactivation (68, 69). It is implicated in SLE, which predominantly affects women and KS males, and is expressed at higher rates in individuals with SLE (69). Lipopolysaccharide, IFN-γ, and IFN-α have been observed to increase CXorf21 expression in monocytes and B cells, suggesting possible roles in both innate and adaptive immunity (68). Specifically, CXorf21 may collaborate with TLR7 to maintain optimal lysosomal pH levels for degradation of pathogenic material, which APC display for T-cell recognition. TLR7 promotes CXorf21 expression, while CXorf21 decreases TLR7 transcription through a feedforward response. As a result, CXorf21 likely participates in the TLR7 pathway and may engage in the SARS-CoV-2 immune response. While its exact function is presently unknown, its overexpression in female APC suggests that CXorf21 may cooperate with TLR7 to contribute to the heightened antiviral response in women (68).

#### CD40L

The gene locus for CD40L has been identified as Xq26.3-27.1 (65). CD40L functions in several aspects of the adaptive immune response, including T-cell differentiation, immunoglobulin class switching, and formation of long-lived plasma cells and memory B cells (70). In particular, CD40L signaling acts on CD8 + T cells to mobilize the mucosal cytotoxic T lymphocyte (CTL) response to viral infection (71). A comparison of antigen presenting cells (APCs) from TS women, KS men, and individuals with typical karyotype found that cells from typical women expressed significantly more CD40L than those from typical men or TS women (65). Klinefelter Syndrome men yielded similar results to women possessing an XX karyotype, suggesting that XCI escape may confer an advantage against viral infections by increasing CD40L expression. Increased CD40L in individuals with an additional chromosome may cause greater T- and B-cell activation, leading to better ability to fight off viral infection.

#### Complement C4

Evidence suggests that the complement system may serve as the first line of defense against SARS-CoV infection. Mannose-binding lectin was found to be depleted in patients with SARS-CoV, suggesting that the complement system may aid in the response to coronavirus infection (72). The presence of mannose binding lectin similarly amplified the binding of complement C4 on SARS-CoV *in vitro*, supporting the activation of the lectin pathway in the SARS-CoV response. C4, which is located at the MHC, is of particular interest because of observed differences between sexes (22). C4 protein is more abundant in the cerebrospinal fluid and plasma of men compared to women, with a greater difference observed in men and women of childbearing age (20–50 years). Moreover, mutation in the C4 gene affects disease risk differently in men and women. Mutations which increase expression of C4 correlate with elevated risk of schizophrenia, SLE, and Sjogren’s syndrome. Women have higher incidence rates of SLE and Sjogren’s syndrome but lower incidence rates of schizophrenia, while the opposite is true for men. Moreover, the sex-based difference in disease incidence mirrors that of C4 protein levels and is most noticeable between men and women aged 20–50. As a result, the discrepancy in disease rates may be attributed to variable effects of C4 between men and women. The different actions of C4 in men and women may contribute to observed disparities in severity and incidence of SARS-CoV among sexes.

#### Human Leukocyte Antigen

Human leukocyte antigen (HLA) is located in the MHC gene group along with C4 and has been linked to AD, including type 1 diabetes and rheumatoid arthritis (22). Human leukocyte antigen enables differentiation between host cells and pathogens through antigen presentation to the T-cell receptor (TCR). The HLA system shapes the TCR repertoire by bolstering or suppressing different T cell lines based on antigen exposure, and thus may affect the adaptive immune response to SARS-CoV-2.

Biological sex has been shown to affect HLA interaction with the TCR. In men with AD (MS and rheumatoid arthritis), CD8+ T cell clonal expansion is less dependent on HLA binding affinity when compared to women with AD, leading to greater production of T-cell clones with low TCR-MHC affinity in men (73). As MHC binding is required for effective T-cell activity, this finding is consistent with the observation that men are biased toward infections and non-reproductive system cancers (hypoactive T-cell response) while women are biased toward autoimmune disorders (hyperactive T-cell response). The sex-based difference in HLA signaling may thus contribute to sex-based differences in COVID-19.

#### NF-κB Essential Modulator

NF-κB essential modulator (NEMO), gene locus Xq28, serves as an activator of the NF-κB pathway (74). As the NF-κB pathway has been implicated in the response to SARS-CoV, NEMO may play a part in warding off SARS-CoV-2 infection (75). Evidence suggests that “one hit inactivation” may disproportionately place men at increased risk of complications from mutated NEMO due to the absence of cellular mosaicism (76). For instance, incontinentia pigmenti, caused by mutations in NEMO, causes lethality in men but has variable presentation in women. Skewed X-inactivation favoring the wildtype allele has been observed in heterozygous women, which supports the notion that X inactivation may confer a protective advantage against immunodeficiency disorders (77). Moreover, men with KS have been observed to escape lethality from incontinentia pigmenti (78). As a result, the presence of an additional X chromosome in women and KS men may decrease the likelihood of NEMO dysfunction and serve as an advantage in the SARS-CoV-2 response.

#### FoxP3

FoxP3 has been mapped to locus Xp11.23 (NCBI FOXP3). Mosaic expression of FoxP3 suggests decreased risk of non-functional FoxP3 in women, compared to men who possess one allele and experience “one hit inactivation” (58). Immune dysregulation polyendocrinopathy enteropathy X-linked syndrome (IPEX), caused by mutant FoxP3, primarily affects men. Because FoxP3 does not undergo skewed X inactivation, heterozygous women express both mutant and wildtype FoxP3 alleles equally (79). However, women heterozygous for mutant FoxP3 exhibit normal lymphocyte levels and normal immune response to infection. The participation of FoxP3 in positive feedback loops, in which FoxP3 protein further stimulates transcription of the FoxP3 gene, indicates that one functional copy of the gene may be sufficient to maintain appropriate levels of FoxP3 (80). As FoxP3 is critical to Treg-mediated immunosuppression, the protective effect of mosaicism implies an immunologic advantage for women at a population level (81). Low levels of Tregs have been associated with increased mortality rates in murine coronavirus-infected mice, while mice administered CD4+CD25+ Tregs yielded decreased mortality rates (40, 82). As a result, the ability to curb excessive activity by cytotoxic neutrophils, macrophages, and other immune cells may decrease risk of fatality from SARS-CoV and other coronaviruses (83). Although Tregs may offer benefit by reducing excessive tissue damage, they may also dampen the immune system and limit ability to clear an infection, indicating the need to strike a balance between the two. While Tregs may be less important in acute viral infections requiring an aggressive immune response, the disease characteristics for SARS-CoV-2 suggest that Tregs may play a crucial role in the antiviral response. Cellular mosaicism and the resulting improvement in genetic diversity may allow women to strike this balance more easily.

### MicroRNA

MicroRNA (miRNA) are short, single stranded non-coding RNA that bind complementary sequences on target genes and block mRNA translation and degradation. Roughly 14% of all miRNA show a sex-biased expression pattern (84). There are 113 miRNA on the X chromosome and 2 miRNA on the Y chromosome (4). Many of these X-linked miRNAs target immuno-suppressive genes like FoxP3, CTLA4, CBL, and SOCS, preventing their translation or triggering their degradation. Given that women have two copies of the X chromosome, and that some of these genes may escape X-inactivation, this may help to explain the sex bias in immune responses. Additionally, it has been found that miRNA that are evolutionarily conserved are more often implicated in disease states (85), and male-specific miRNA evolve more quickly than female miRNA (84) and therefore are less conserved. Taken together, this suggests that female-specific miRNA have more pro-inflammatory effects than male-specific miRNA.

Several studies have shown the role of miRNA to control the host cell response to infections by RNA viruses and to control the virus’ levels of infectivity via binding to viral RNA (86, 87). Following the binding, the miRNA can either inhibit translation and decrease viral infectivity or it can stabilize the RNA and effectively increase translation. These studies have shown miRNA interaction with RNA viruses such as Hepatitis C Virus (HCV), Human Immunodeficiency Virus (HIV), West Nile Virus (WNV), Dengue Virus (DV), and Influenza A Virus (IAV). In contrast, the viral RNA itself can have an impact on host miRNA to help evade the host immune system. This is exactly what was observed in the MERS and SARS-CoV infections. An *in silico* gene expression analysis (87) revealed that SARS-CoV upregulates miRNA-17, -574-5p, and -214 and MERS upregulates miRNA-628-5p, -6804-3p, -4289, -208a-3p, -510-3p, -18a-3p, -329-3p, -548-ax, -3934-5p, -4474-5p, -7974, -6868-5p, and -342-3p. Together, these contribute to viral evasion of the immune response. This data suggests that the novel SARS-CoV-2 virus may respond in a similar fashion and raise the possibility of miRNA as a therapeutic target.

### Sex Hormones

Sex hormones are an important biological factor contributing to the gender-bias in the immune response, and can influence outcomes of disease severity in infections and autoimmunity (4, 88–90). Variations in sex hormone levels throughout the lifespan as with puberty, pregnancy, exogenous sex-hormone therapies, aging/menopause, and in transgender individuals modulate the immune response to pathogens therefore underlying the importance of their study. In general, estrogens are considered immuno-stimulatory and activate both the innate and adaptive immune responses and therefore women are able to clear pathogens more efficiently than men, whereas testosterone is immuno-suppressive, which may underlie the higher susceptibility and severity of infectious diseases in men (4). On the other hand, the stronger immune response in women is thought to underlie the disproportionately high prevalence of AD in women over men.

Sex hormones control both cellular and humoral components of the immune response and thus determine the sex-bias in susceptibility, manifestations and clinical outcomes in infections, AD and malignancies (4, 90). Immune cells bear estrogen receptors (ER) α and β, progesterone receptors (PR), and androgen receptors (AR), which are ligand-activated transcription factors. Sex hormones bind these receptors and trigger intracellular signaling cascades to regulate gene and protein expression to influence development, maturation, activation, and function of innate and adaptive immune cells during homoestasis and the immune response to infections. While the immune responses are in part mediated against the infectious agent and protective to the host, an overactive response such as overproduction of inflammatory cytokines can lead to severe immunopathology and organ damage and ultimately fatality, as is seen in certain respiratory viruses with complications of ARDS in the lungs during the SARS-CoV, MERS, and the current SARS-CoV-2 COVID-19 pandemic. Better understanding of the factors that control the immune response in a sex-specific manner is therefore crucial to not only understanding disease pathogenesis but also guiding treatment and prevention strategies and a first step toward personalized medicine.

The sex-biased factors that impact immunity have developmental origins beginning *in utero*, infancy and childhood (91). For example, placental hormones help shape fetal and neonatal immunity, and some of these influences are retained through adulthood. Estrogen and progesterone are important in alveolarization and surfactant production respectively. Sex hormones surge in a time of early infancy termed as “mini-puberty”; this influences the immune system and early childhood susceptibility to infections in boys versus girls. For some infections and AD, these differences in susceptibility and severity are retained through adulthood but may change or even reverse for some allergy-related conditions and AD.

Studies on the role of sex hormones in immune cells range from *ex vivo* cultures of human or mouse cells, or *in vivo* supplementation in mice after gonadectomy, including those assessing mice with genetic deletions of sex hormone receptors. Given the wide variations in human versus rodents *in vitro* versus *in vivo* systems, epidemiological studies have shown that there is not a universal paradigm regarding the role of gender or sex hormones on the immune response to respiratory viruses. It is hypothesized that the disease outcomes are ultimately a combination of the magnitude of the immune response and degree of host tissue damage (92, 93). There is a male bias when a weaker immune response contributes to damage, while a female bias may occur due to a stronger immune response that causes damage.

#### Estrogen and Innate Immunity

Estrogen-ER signaling regulates innate myeloid cells including pDCs, monocytes, neutrophils, and lymphoid cells, including innate lymphoid cells (ILC) (16). Estrogen is known to promote type I IFN synthesis, and female plasmacytoid DCs produce more type I IFN in response to viral nucleic acids and TLR-7 activation than males, which correlates with IRF 5 levels. Lower physiologic concentrations of estrogen are known to enhance the proinflammatory cytokines IL-12, IL-6, and IL-1β, while higher physiologic concentrations diminish their levels and in turn promote IL-10 regulatory cytokines (16). Furthermore, estradiol promotes the differentiation of murine BM-derived DCs by increasing IRF4 transcription factor levels. Estrogens contribute to delayed neutrophil apoptosis and can modulate chemotaxis and NO production *in vitro*. The lung-resident alveolar macrophages are important in respiratory infections and produce type I IFN for viral clearance. Although they express both ERα and AR, sex differences or the role of sex hormones on these cells during respiratory viral infections have not been reported (16). Differentiation of M1 and M2 subtypes by a type 1 IFN-driven or a type 2 IL-4/IL-13-driven response are known to be influenced in allergic asthma, where females/ERα promotes the M2 phenotype important for tissue repair. These findings imply that wherein estrogen and ERa enhance while AR may dampen the type 2 responses important for lung tissue repair post-viral infections. ILC2s important in tissue repair and secrete IL-5/IL-13 are the prominent subtype in murine lungs and their numbers are increased in female mice and in humans compared to males. While these cells predominantly express AR, there is tissue specific regulation by sex hormones and estrogen-ER signaling promoted uterine over lung ILC2. Elevated numbers in IAV infections may provide superior tissue repair, however their plasticity to convert to ILC-1 like cells and IFN-g production may make them more pathogenic and contribute to immunopathology (16).

#### Estrogen and Adaptive Immunity

In general, estrogens are immune-stimulatory and are known to be involved in T-cell development, activation, differentiation and function (4). Estrogen-ER signaling was shown to be necessary for normal thymic size and development, and furthermore estrogen is known to promote extrathymic T-cell differentiation in the liver. Its role in T-cell homeostasis with respect to cell survival and proliferation is complex and varies depending on cell type, context, and concentration, where physiologic doses of estradiol suppress apoptosis whereas pharmacologic doses suppress proliferation in cancer cells. Estrogen and ERα promote CD4 T-cell activation and deletion of ERα led to altered transcriptomics with reduced levels of genes involved in T-cell activation. Estrogen controls cell metabolism and genes involved in metabolic activity important to stimulate T-cell differentiation and stimulate mitochondrial function.

Estrogen increases signaling through NK-κB to activate production of inflammatory cytokines including IL-1β, IL-10, and IFN-γ in mouse splenocytes (4). Estrogen is known to suppress IL-2 cytokine production in human T cells (94) and rat splenocytes. Accordingly, lower IL-2 levels are observed during the luteal phase of the menstrual cycle in healthy young women and thought to contribute to the observed increase in pre-menstrual infections. Estrogen is known to increase proliferation of CD4 T cells and increase CD4 Th1 differentiation and IFN-γ cytokine production which are necessary in the cell mediated response to viral infections and in addition increases the inflammatory response mediated by IFN-γ via activation of iNOS, NO, and COX2. CD4 T follicular helper (Tfh) cells are crucial for providing cognate help to B cells and promote class switching and somatic hypermutation to produce antibodies. Estrogen was shown to promote the expression of Calcineurin and CD40L in human T cells (95), molecules important for help to B cells in the antibody response. T cells traffic within the body to peripheral tissue sites of infection and migrate across chemokine gradients via chemokine receptors expressed on their surface. Estrogen promotes both chemokines as well as chemokine receptor expression as evidenced by *ex vivo* and *in vivo* studies in mice (4). Female mice expressed higher levels of chemokine receptors CCR1-CCR5 in response to chemokines and mice administered estrogen expressed increased levels of MCP-1, MCP-5, Eotaxin, and SDF. Estrogens also enhance CD8 T-cell activity and suppress Th17 immune responses.

The role of Tregs in response to viral infections is complex. Estrogen increases FoxP3 levels and Tregs *in vitro* and correlations have been observed *in vivo*. Increased numbers of circulating Treg cells are observed during the late follicular phase of the menstrual cycle in fertile non-pregnant women which dropped in the luteal phase, correlating with β-estradiol levels. In women with recurrent spontaneous abortions (RSA), lower Treg levels were found in both follicular and luteal phases and in postmenopausal women. The suppressive capacity of these Tregs was also lower in case of RSA.

Estrogen promotes B-cell homeostasis, activation, maturation, and differentiation and enhances immunoglobulin production (4). These properties make women and female mice able to mount greater magnitudes of neutralizing antibody responses to infections and thus contribute to protection against respiratory viral infections including the SARS-CoV infections. Estrogen administration led to increased marginal zone B cells in the spleen and in transgenic mice led to elevated anti-dsDNA antibodies. Estrogen promoted the expansion of high-affinity antibody-producing B cells and also promoted survival by increasing expression of the Bcl-2 anti-apoptotic molecule (96). In addition, higher levels of B lymphocyte stimulator (Blys) also called B cell activating factor (BAFF) were observed in female C57Bl/6 mice. Estrogen administration elevated BAFF levels and this was reverted in mice deficient in ERα, STAT1, or IRF5 (97). Besides controlling B-cell activation which enhances the Ig antibody response, ER can directly control the Ig as ER-binding ERE have been found within the heavy chain locus of Ig by an ERα antibody-mediated ChIP-sequencing analysis of genomic DNA (4).

#### Progesterone and Androgens

Immune cells express PR and AR, and progesterone, androgen, and testosterone in particular are considered immuno-suppressive and may counteract the effects of estrogens, contributing to the observed increased susceptibility to the SARS-CoV-2 and disease in men (98–100). Progesterone downregulates IL-1β and TNF proinflammatory cytokine production by BM-DCs in mice, and testosterone is known to decrease cytokines including IFN-γ and TNF and increase regulatory cytokines such as IL-10. Androgen receptor-deficient mice exhibit reduced numbers of neutrophils and accordingly increased susceptibility of male mice to SARS-CoV infection correlated with accumulation of neutrophils in the lung. Progesterone reduces T-cell proliferation and T-cell-dependent antibody responses in human peripheral blood and cell line or mouse studies. Furthermore, progesterone regulates CD4 Th differentiation and cytokine production with increased IL-4, increased Treg cell differentiation, and reduced IFN-γ, Th17 responses. In cytotoxic CD8 T cells, progesterone reduces IFN-γ and cytotoxicity. Its effects on B cells included reduced class switch recombination and reduced T cell dependent antibody production. Normal testosterone levels are associated with normal respiratory capacity, whereas plasma testosterone levels decline in men with increasing age with observed associations between an increase of pro-inflammatory states and decline in testosterone in aging men. Furthermore, hypogonadism is associated with elevated pro-inflammatory cytokines and testosterone decreases levels of IL-1β, IL-6, and TNF-α (98). On the other hand, high androgen levels may promote or contribute to infection because AR mediated transcription of TMPRSS2 protease which is important for viral entry into host cells.

A proposed androgen sensitivity model provides a link between increased disease severity in men and the role of androgens in COVID-19 (101). Androgen sensitivity is primarily determined by genetic variants of the AR. Specifically, shorter CAG repeat polymorphisms in the AR gene have been associated with both androgen sensitivity and more severe COVID-19 symptoms. Shorter CAG repeat polymorphisms may cause overexpression of the transmembrane protease, serine 2 (TMPRSS2) gene due to greater activation of the AR. TMPRSS2 overexpression in turn may enable greater viral entry and replication through cleavage of both the SARS-CoV-2 spike protein and ACE2 receptor, facilitating viral uptake and virus-cell fusion (Wambier). In addition, it has been observed that SARS-CoV cleavage by TMPRSS2 releases spike protein fragments that act as decoys for neutralizing antibodies (102). A similar mechanism may function in SARS-CoV-2 infection to dampen humoral immunity. As TMPRSS2 transcription is activated upon AR binding, elevated TMPRSS2 in men and individuals with high androgen levels may contribute to sex-based disparities in COVID-19 severity through the following mechanisms: viral replication promotion and humoral immunity suppression.

While men are generally predisposed to these effects due to higher testosterone levels compared to women, individuals with hyperandrogenism and other conditions may similarly be impacted. For example, women with hirsutism and polycystic ovarian syndrome (PCOS), as well as women taking progesterone-based birth control, may be at greater risk for more severe COVID-19 symptoms (101). Markers of androgen sensitivity such as PCOS, androgenetic alopecia, and prostatic hyperplasia may thus be used as clinical signs of vulnerability. The high rates of androgenetic alopecia observed in hospitalized COVID-19 patients supports the theory that elevated androgens may contribute to more severe COVID-19 symptoms by increasing TMPRSS2 expression in target tissues.

#### Clinical Trials With Estrogen and Progesterone for COVID-19

In this pandemic, men are more acutely ill and exhibit higher death rates with disproportionately higher numbers in ICU and requiring ventilators compared to women. Even pregnant women had lower rates and less severe disease and complications than men. Estrogen and progesterone levels rise exponentially during pregnancy, causing a shift in the immune response, and this may underlie the observed protective effects. The importance of these female sex hormones in the immune response to infections has triggered two clinical trials with sex hormone administration to patients with COVID-19. One trial at the Renaissance School of Medicine Stony Brook University, Long Island, New York, is administering Estrogen to 110 adult patients, including men and women over 55, who present to the ER with symptoms of COVID-19 like fever, cough, shortness of breath or pneumonia. Half will receive a single-use transdermal estradiol patch for 7 days, and the other half will serve as a control group and receive standard of care. The second, smaller randomized controlled trial with 40 male patients at Cedars-Sinai hospital in Los Angeles will administer progesterone in an effort to suppress the overactive immune response and mitigate immunopathology. Inpatients with mild to moderate disease will be included and half will receive progesterone 100 mg subcutaneous twice daily for 5 days, and the other half is a control group. Since progesterone is immune-suppressive and diminishes the proinflammatory response, this trial is intended to determine whether progesterone treatment can reduce the incidence of cytokine storm and related immunopathology leading to ARDS.

### Microbiome

It is well-known that the commensal bacteria in the GI tract impact the immune response. Some possible mechanisms involve microbiota affecting and regulating cytokine production (103), while others involve microbiota modulation of the production of mucous and antiviral defensins and ROS (104). In regard to viral infections, however, some microbiota elicit protective effects, while others serve as a route of viral entry and infection. For example, the Lactobacillus genus prevents murine norovirus replication *in vitro*, and there is *in vivo* evidence that this genus is decreased in a mouse that is affected by norovirus. In response to Influenza and WNV, gut microbiota secrete IgA and upregulate TLR-7 in the respiratory mucosa (105) in order to promote activation of important components of antiviral immunity—cytotoxic T lymphocytes, Th1 cells, and inflammasomes. On the other hand, human and murine norovirus utilize the commensal gut bacteria as a place to harbor and/or break through the mucosa—leading to infection.

There is currently less known about the interaction between SARS-CoV-2 and gut commensal bacteria, but it has been noted that COVID-19 patient fecal samples contain higher numbers of the Prevotella genus (106). It is therefore possible that there is an interaction between these bacteria and the virus, though the exact relationship is unknown. One possible mechanism is that, similarly to the human norovirus, the normally commensal bacteria are harboring the virus and are producing a cytokine response that is inappropriate for the response to the infection. This ultimately would result in dysbiosis and a worse outcome for the patient. Alternatively, a relationship between ACE-2 and the gut microbiome may play a role in the immune response. SARS-CoV and SARS-CoV-2 bind to the ACE-2 receptor and cause downregulation. This protein is critical for the transportation of tryptophan across the epithelium, which then normally increases the production of antimicrobial peptides that affect the composition of the microbiome (107). Lack of this peptide production would likely result in dysbiosis and an impaired immune response.

Recently, it has been noted that sex hormones have a large effect on the microbiome. Particularly, higher levels of systemic estrogen, like those seen in women, are positively associated with the richness and diversity of the fecal microbiome (108). Additionally, germ-free female mice have higher baseline antibody levels than germ-free male mice (109). Women have higher expression of antiviral and pro-inflammatory genes (92), and male mice show higher levels of the anti-inflammatory molecules TGF-β, IL-10, and FoxP3. Additionally, male mice who received an *in vivo* transplant of female microbiota showed increased T cell precursors in the thymus and decreased levels of RORyt and FoxP3 + cells (110). RORγt cells are important for controlling the Th17 cell response, while FoxP3 + cells are Tregs that help to control the immune response. Taken together, these studies suggest that female sex hormones, particularly estrogen, have a pro-inflammatory effect (4) that promotes a more robust response to infection.

In addition to lacking the stimulatory effects of estrogen, men also produce androgens that seem to have a protective mechanism against the immune response. Testosterone is known to decrease the ability of NK cells to produce and secrete IFN-γ, an important mediator of the immune response (111). Men also have lower CD3+, CD4+ counts, lower CD4+/CD8+ ratios, and lower Th1 responses than women (92). Furthermore, the testosterone surge at puberty in male mice dampens B- and T-cell development (112).

Commensal bacteria in the gastrointestinal tract have a role in regulation of testosterone levels (109). An *in vivo* study found that the number of species in microbiomes of mice was not significantly different during the pre-pubescent stage but was significantly different following puberty (113). This suggests that hormone changes during puberty drive changes in the microbiome. Further, the microbiome elevates androgens to a level that confers protection from type 1 diabetes in mice (112, 113), which illustrates the synergistic effect of the male hormones and the microbiome. While this is thought to be a major factor in the protection against autoimmunity, it is also reasonable to think that the immune response may be dampened below the level that is needed for a strong response to pathogens. Overall, these findings suggest that the microbiome is an important biological factor in the sex-bias in response to infection and may be involved in the SARS-CoV-2 responses.

### ACE2

The SARS-CoV-2 spike (S) glycoprotein enters the host cell via binding to the ACE2 receptor. This binding leads to the subsequent downregulation of ACE2, which is considered to be protective against lung injury. ACE2 downregulation leads to pathway shunting toward ACE enzyme and a buildup of angiotensin II. Stimulation of AGTR1a receptor by angiotensin II leads to endothelial cell permeability which may explain the increase in pulmonary pathology with decreasing levels of expressed ACE2.

The location of ACE2 on the X chromosome suggests possible genetic influence in the elevated male mortality rate. As the binding affinity between SARS-CoV-2S protein and ACE2 dictates transmissibility and disease severity, the presence of two cell lines and subsequently two ACE2 variants in women may contribute to the sex-based differences observed in COVID-19 patients. For example, mutations in ACE2 in one cell line may alter the catalytic site and lead to divergent viral susceptibilities between cell populations decreasing peak viral load (114). Notable protein-protein interactions between ACE2 and SARS-CoV-2 include a salt bridge formation between Lys317 of the SARS-CoV-2 receptor binding domain (RBD) and Asp30 of ACE2 as well as a Van der Waals interaction between Leu472 of the RBD and Met82 of ACE2 (115). Substitutions in these amino acids, among other mutations, may alter binding affinity between the RBD and receptor, limiting the ability of the virus to enter the cell and propagate.

Variable expression of ACE2 may also influence patient outcomes. Viral entry into a cell causes downregulation of ACE2, which may be detrimental in patients already deficient in ACE2 (116). ACE2 downregulation leads to pulmonary edema, alveolus hyalinization, and leukocyte accumulation. Cecal ligation and perforation of ACE2 knockout mice has also been shown to increase lung failure and tissue damage, indicating that ACE2 may confer a protective role in microbial infection (117). Gene dosage, however, likely does not contribute to the sex-based difference in SARS-CoV-2 response, as evidence suggests that sexual dimorphism in ACE2 expression persists in renal tissue but not cardiac or pulmonary tissue under non-pathological conditions (118). Moreover, changes in chromosome dosage was not observed to affect ACE2 expression in mice that had undergone gonadorectomy. This finding implies possible hormonal but not chromosomal effects in ACE2 expression levels.

Despite limited evidence supporting the effect of chromosome dosage in the increased male mortality rate, cellular mosaicism in women may offer protection against immune deficiency. As males are hemizygous for ACE2, they experience “one hit inactivation” due to the absence of the “back-up” allele present in women. As a result, the effect of detrimental mutations to ACE2 may be more pronounced in men than women, altering the clinical course in male versus female patient populations.

The ACE2 receptor is expressed in the type II pneumocytes of the lungs and also in other tissues, including the heart, tubular epithelial cells in kidneys, testis, adipose tissue, and the enterocytes in the gastrointestinal tract and vascular endothelial cells (119). A recent study evaluated ACE2 expression in older men and women with heart failure and found in two independent cohorts that circulating plasma concentrations of ACE2 were higher in men than in women (120). This may reflect differences in tissues from men versus women. Two studies utilized systems biology approaches of meta-analysis, co-expression and network analysis to draw information on the expression, regulation and gender bias of ACE2 receptor expression. One study (121) evaluated datasets from the Genotype-Tissue Expression (GTEx) project, the cancer genome atlas (TCGA) program and the human protein atlas (HPA) database to evaluate ACE2 expression in 31 normal human tissues, comparing men versus women and younger (ages = 49 years) versus older (ages = 49 years) individuals and further correlating ACE2 expression with immune signature enrichment (CD8+ T cells, IFN response, B cells, and NK cells) across tissues. The highest levels of ACE2 expression were found in the small intestine, testis, kidneys, heart, thyroid, and adipose tissue, medium levels in the lungs, colon, liver, bladder, and adrenal gland, and lowest in blood, spleen, bone marrow, brain, blood vessels, and muscle. While they did not find a significant difference in gene expression between men and women, ACE2 expression in the lungs was positively correlated with immune signatures in men and negatively in women. In addition the HPA database showed high levels of both ACE2 gene and protein in the gastrointestinal tract (duodenum, small intestine, colon, and rectum), kidney, gallbladder, and male tissues (testis and seminal vesicle). Taken together these data suggest that the differential host immune responses may underlie the gender-bias of the remarkably distinct clinical outcomes. The other study of patients with severe COVID-19 who had comorbidities (122) evaluated data from over 700 lung transcriptome samples and found that ACE2 was highly expressed in these patients, compared to controls. Correlation and network analyses revealed many histone-related genes HAT1, HDAC2, and KDM5B. Suggesting epigenetic regulation of ACE2 in the human lung.

ACE2 is known to be expressed in Leydig cells of both mice and humans, albeit testosterone-independent, and is thought to contribute to steroid synthesis (123, 124). It is also expressed in ovarian granulosa cells and its levels increase in correlation with increasing LH levels. In addition to expression in the gonads, ACE2 expression and activity is influenced by sex hormones in adipose tissue, myocardium, and kidneys. ACE and ACE2 are intricately involved in the renin angiotensin system for fluid/electrolyte balance and cardiovascular homeostasis and control of metabolic factors contributing to obesity, hypertension and related cardiovascular complications. One study examined the role of sex hormones on cardiac ACE and ACE2 activity. Higher ACE and ACE2 activity and cardiac hypertrophy was found in male rats compared to female rats which was reduced after orchiectomy, while ovariectomy elevated ACE2 and hypertrophy in females (125). In male mice, chronic high fat diet (HFD) led to decreased ACE2 expression in the kidneys. In female mice, HFD increased adipose tissue ACE2 which was reversed by ovariectomy implying that estrogen increases ACE2 expression and activity in adipose tissue and kidneys. Furthermore global deficiency of the ACE2 gene increased HFD-induced obesity hypertension in male mice (126, 127). Importantly, ovariectomy or treatment with an ER antagonist in SARS-CoV infected female mice increased the mortality rate therefore, suggesting a protective effect for the ER signaling pathway in mice (3).

While sex hormones influence ACE2 expression and activity to influence outcomes in obesity, hypertension, and related comorbidities, thus influencing COVID-19 outcomes, the effect of COVID-19 on male sex hormones has been recently explored. Given that the ACE2 receptor is expressed in the testes, a study from Hubei province of China reports that the COVID-19 impacts male gonadal function and observed alterations in hormone levels. They studied 81 men with COVID-19 and found that serum luteinizing hormone (LH) levels were increased while the ratio of testosterone to LH and the ratio of follicle stimulating hormone (FSH) to LH were significantly lower compared with 100 age-matched healthy men (128). Recent reports of increased frequency of venous thromboembolism, associated with worse outcomes in patients with COVID-19 warrant caution in treatment with testosterone, specifically in hypogonadal men with greater genetic predisposition.

### Immune Response to Vaccines

Besides its role in infection immunity, sex is equally important in the immune response to vaccines (129, 130). Women not only mount stronger antibody and T-cell responses to vaccinations than men, but also suffer more adverse events. Yet there is a serious lack of attention to gender in vaccine trials. This leads to inappropriate dosage of vaccines as evidenced by the fact that the same magnitude of protective immunity is achieved by half the dose of seasonal influenza vaccine in women compared to men. Likewise, these gender-blind vaccination strategies lead to increased adverse effects in women. Increased hospitalizations and mortality have been observed in female infants and girls following DPT, measles and oral polio vaccinations (130). Sex-based biological factors include differences across the immune system within innate immunity, antibody responses and T cell responses. Genetics, sex hormones, epigenetic factors, nutrition, and the microbiome are important biological contributors to these sex-based differences. Vaccine-related research and clinical trials, including those currently underway for COVID-19, must thus include sex as a key variable when measuring and reporting outcomes of immunogenicity and reactogenicity. This information would help tailor vaccine dosage and strategies appropriately to maximize protective immunity while minimizing adverse effects.

## Conclusion

The COVID-19 pandemic has revealed a striking gender-bias with increased case and mortality rates in men compared with women across the lifespan. Besides behavioral and lifestyle factors, sex-based physiological differences influence the host immune response to infections. Sex chromosome linked genes, sex hormones, and the microbiome control aspects of the innate and adaptive immune responses to infection. Genetics and sex hormones also regulate and influence aspects of viral entry including expression and activity of ACE2 and TMPRSS2. These differences not only affect the risk/susceptibility to infection but also the disease course/clinical outcomes and response/adverse effects to vaccines. Better understanding of these factors is necessary to tailor therapies and vaccine strategies in a step toward sex-based personalized medicine.

## Author Contributions

VM conceptualized the article. All authors contributed to the literature review, writing, and finalizing the manuscript.

## Conflict of Interest

The authors declare that the research was conducted in the absence of any commercial or financial relationships that could be construed as a potential conflict of interest.

## Footnotes

1 https://covidprotocols.org
